# Prevalence of Smoking Among Health Science Students in the United Arab Emirates

**DOI:** 10.7759/cureus.107304

**Published:** 2026-04-18

**Authors:** Tala N Almaliti, Suhaib M Dweik, Abbas A Atraqchi, Hind A Almaeeni, Zainab F Alqallaf, Merna R Abdelwahed, Donea N Almaliti, Laelas Barnia, Basema Saddik, Amal Hussein

**Affiliations:** 1 Department of Clinical Sciences, College of Medicine, University of Sharjah, Sharjah, ARE; 2 College of Medicine and Health Sciences, Khalifa University, Abu Dhabi, ARE; 3 Department of Family and Community Medicine and Behavioral Sciences, College of Medicine, University of Sharjah, Sharjah, ARE

**Keywords:** electronic cigarettes' e-cigarettes' vaping' e-smoking, health science students, nicotine dependence, smoking habits, smoking prevalence, tobacco products, uae

## Abstract

Introduction

In the United Arab Emirates (UAE), tobacco consumption has increased markedly in recent years, as rapid economic and social changes have led to a rise in the popularity of tobacco use. Since healthcare workers are on the front lines in combating tobacco use and abuse, it is vital that aspiring health science students are aware that their own smoking status can be an important determinant of their ability to help patients control tobacco use.

Aim

The purpose of this study is to determine the prevalence of smoking among health science students in the UAE.

Methods

A cross-sectional study was conducted among health science students aged 18 years and older from multiple universities across the UAE between February and June 2023. Participants were recruited using convenience snowball sampling through university networks and social media platforms. Data were collected using a structured bilingual (English/Arabic) online questionnaire assessing sociodemographic characteristics, smoking behavior, factors influencing smoking initiation, awareness of smoking-related harms, and nicotine dependence. Nicotine dependence among current smokers was measured using the Fagerström Test for Nicotine Dependence (FTND).

Results

A total of 406 students participated (31.8% male, 68.2% female). The overall smoking prevalence was 20.4%, including 15.0% current smokers and 5.4% ex-smokers. Smoking prevalence was significantly higher among males compared to females (32.6% vs 14.8%, p = 0.011). The most commonly reported reasons for smoking were stress (43.4%) and pleasure (32.5%). Male smokers demonstrated significantly higher nicotine dependence scores compared to females (p < 0.001). Additionally, smokers using multiple tobacco products had significantly higher FTND scores (p = 0.030). While general awareness of smoking-related health risks was high, gaps and misconceptions were identified.

Conclusion

Intervention strategies targeting stress management among health science students may be beneficial; however, causal relationships cannot be established due to the cross-sectional design. Nationwide studies are required to obtain findings that are more generalizable to the population.

## Introduction

Smoking is defined as the act of drawing smoke from burning tobacco into the mouth and, usually, the lungs [1]. It can be practiced using nicotine-containing substances, such as cigarettes, cigars, waterpipes (shisha), midwakh (dokha), or electronic nicotine delivery systems (ENDS) [2]. Globally, tobacco use remains a leading preventable cause of disease, disability, and premature mortality. The WHO recognizes tobacco use as an epidemic, responsible for more than eight million deaths every year [3]. It creates a significant burden on the economy and public health and is a major contributor to cardiovascular disease, chronic obstructive pulmonary disease, stroke, and various cancers, including those of the lung, larynx, bladder, and oral cavity [3].

In the United Arab Emirates (UAE), recent studies on university students, including those in health-related disciplines, reveal a continued prevalence of tobacco consumption. Multi-university studies report that 15.1% of students currently use tobacco, with 7.2% using midwakh and around 4% using e-cigarettes [4]. Midwakh has been recognized as an emerging public health issue [5]. A recent multi-country study found that vaping is now the most prevalent tobacco product (21.2%) among Arab university students [6]. Another study of students in Dubai found that around 16% smoke cigarettes and approximately 26% use waterpipes, with a higher rate among males [7]. Studies conducted at the University of Sharjah [8] found that 28.2% of students smoked, primarily using cigarettes, midwakh, and waterpipe, with a noted dependency on shisha among 16.3% [4,7,9].

These findings highlight not only the persistence of tobacco and nicotine use among university students in the UAE, but also the diversity of product use and the upward trend in alternative nicotine delivery systems such as waterpipes and ENDS [4,7].

Of particular concern is the prevalence of smoking among health science students, who represent the future healthcare workforce. A recent study in 2025, aiming to assess the prevalence of unhealthy lifestyle behaviors, revealed that 23.8% of health science students in Ajman smoke [10]. This habit not only endangers their personal health but can also adversely affect their future ability to provide effective tobacco cessation counseling to patients [11]. Furthermore, studies conclude that smoking and nicotine dependence are predictive of poorer academic performance, including lower grade point averages and higher absenteeism among medical students, which can compromise their professional training [12]. Therefore, addressing the prevalence of smoking within this group is critical to ensuring a professional healthcare workforce capable of engaging in tobacco control efforts.

While previous studies have examined smoking among university students in the UAE, there remains limited multi-university data specifically focusing on health science students. This underscores the significance of our study, which aims to fill an existing gap in the literature and provide new insights into the prevalence and determinants of smoking among health science students across the UAE. Therefore, the main aim of this study is to measure the prevalence of smoking among health science students in the UAE. In addition, it aims to identify patterns of smoking, causes of initiation, and types of smoking among these students. The specific objectives are to determine the patterns of smoking among health science students, identify the causes of smoking initiation, assess their awareness of the harmful consequences of smoking, and evaluate nicotine dependence among students who smoke.

The findings from this study will provide crucial evidence to inform future public health interventions and shape targeted strategies to address this issue within UAE health science institutions.

## Materials and methods

Study design and setting

A cross-sectional study was conducted among health science students enrolled at various universities across the UAE. Data collection took place between February 2023 and June 2023. The minimum required sample size was calculated to be 385 participants using the Raosoft sample size calculator, assuming a 95% confidence level, a 5% margin of error, and a response distribution of 50%. To account for potential non-response and incomplete data, the final sample size was increased to 406 participants.

Study population and sampling

The target population consisted of undergraduate health science students studying at universities in the UAE. Students were eligible for inclusion if they were currently enrolled in a health science program at a university in the UAE, were aged 18 years or older, and were able to complete the questionnaire in either English or Arabic. Both smokers and non-smokers were included to allow estimation of smoking prevalence within the study population.

Participants were recruited using a convenience snowball sampling approach. The survey link was initially distributed through university networks, student groups, class representatives, and social media platforms commonly used by students, including WhatsApp. Participants were encouraged to share the survey with other eligible health science students within their networks. Responses were screened for completeness prior to analysis, and incomplete questionnaires were excluded.

Participants were categorized based on self-reported smoking behavior as non-smokers, social smokers, ex-smokers, or current smokers. A current smoker was defined as an individual who reported regular smoking of any tobacco or nicotine-containing product within the past 30 days. A social smoker was defined as an individual who reported occasional or non-daily smoking. An ex-smoker was defined as an individual who previously smoked but was not currently smoking. A non-smoker was defined as an individual who had never smoked.

For the purpose of prevalence estimation, participants categorized as social smokers (occasional or non-daily users) were excluded from the prevalence calculation to avoid overlap with the definition of current smokers. Smoking prevalence was therefore calculated based on current smokers and ex-smokers only.

Sample size 

The minimum required sample size was calculated using the Raosoft sample size calculator, assuming a 95% confidence level, a 5% margin of error, and a response distribution of 50%. Based on these assumptions, the minimum required sample size was 385 participants. A total of 406 completed responses were included in the final analysis.

Data collection tool 

Data were collected using a structured, self-administered questionnaire developed in Google Forms. The questionnaire consisted of five sections: (1) sociodemographic characteristics, (2) smoking behavior and patterns, (3) factors influencing smoking initiation, (4) awareness of smoking-related harms, and (5) nicotine dependence.

The questionnaire was reviewed by faculty members with expertise in public health and/or epidemiology to assess the content validity, clarity, and relevance of the items. A pilot test was conducted among health science students to evaluate comprehension, wording, and completion time. Based on the feedback obtained, minor modifications were made to improve clarity and wording. Participants included in the pilot test were excluded from the final analysis. The questionnaire was made available in both English and Arabic. The Arabic version was reviewed to ensure clarity and linguistic appropriateness before distribution.

Nicotine dependence among current smokers was assessed using the Fagerström Test for Nicotine Dependence (FTND) [13], a validated six-item instrument widely used to assess the intensity of physical dependence on nicotine. FTND scores were categorized as follows: 0-2 (very low dependence), 3-4 (low dependence), 5 (moderate dependence), 6-7 (high dependence), and 8-10 (very high dependence).

Data analysis

Data were entered and analyzed using IBM SPSS Statistics version 28. Descriptive statistics were used to summarize participant characteristics and study variables. Categorical variables were presented as frequencies and percentages. Continuous variables were summarized using appropriate measures of central tendency and dispersion according to data distribution.

Associations between categorical variables were assessed using the Chi-square test. For non-normally distributed continuous variables, the Mann-Whitney U test and Kruskal-Wallis test were used, as appropriate. A p-value of <0.05 was considered statistically significant.

Ethical considerations 

Ethical approval for this study was obtained from the Research Ethics Committee of the University of Sharjah (REC-23-01-29-01). Participation was voluntary, and electronic informed consent was obtained from all participants prior to questionnaire completion. No identifying personal information was collected, and confidentiality was maintained throughout the study.

## Results

Participant characteristics

A total of 406 health science students from various universities across the UAE participated in the study. All respondents were 18 years of age or older, with a mean age of 21.4 ± 2.1 years. The sample included 129 males (31.8%) and 277 females (68.2%). More than half of the participants were medical students (55%), while the remaining 45% were enrolled in other health science programs, including pharmacy, physiotherapy, and dentistry. Regarding living arrangements, the majority of respondents (75%) reported living with their families, whereas the remaining participants resided in university dormitories or independent accommodation.

Prevalence of smoking

As shown in Table 1, among the total participants (N = 406), 15.0% (n = 61) were current smokers, 5.4% (n = 22) were ex-smokers, 8.4% (n = 34) were social smokers, and 71.2% (n = 289) were non-smokers. For the purpose of prevalence estimation, smoking prevalence was calculated based on current smokers and ex-smokers only, giving an overall prevalence of 20.4% (n = 83). Smoking prevalence was significantly higher among male students (32.6%) compared to female students (14.8%), with the difference reaching statistical significance (Chi-square test, p = 0.011).

**Table 1:** Prevalence of smoking among health science students (N = 406).

Smoking status	Frequency (n)	Percentage (%)
Non-smoker	289	71.2
Social smoker	34	8.4
Ex-smoker	22	5.4
Current smoker	61	15
Overall smoking prevalence (current + ex-smokers)	83	20.4

Smoking habits and reasons among smokers

As shown in Table 2, the association between reasons for smoking and selected characteristics among smokers (n = 83) was examined. Stress was the most commonly reported reason for smoking (43.4%), followed by pleasure (32.5%) and peer pressure (24.1%). When examining the age of smoking initiation, an equal proportion of smokers who reported stress as the main reason began smoking before and after the age of 18 (50.0% each), while those who reported pleasure as their main motivation were more likely to initiate smoking before the age of 18 (66.7%). However, no statistically significant association was observed between age of initiation and reason for smoking (χ²(2) = 2.628, p = 0.269). Similarly, no statistically significant association was found between year of study and reason for smoking (χ²(2) = 1.113, p = 0.573). Regarding social influences, the majority of smokers reported having a smoking partner, particularly among those who cited peer pressure as their reason for smoking; however, this association approached statistical significance (χ²(2) = 5.858, p = 0.053). Additionally, no statistically significant association was observed between tobacco use in the household and the reported reason for smoking (χ²(2) = 4.077, p = 0.130). Overall, these findings suggest that while stress is the predominant reason for smoking among health science students, the demographic and environmental factors examined in this study were not significantly associated with the reported motivations for smoking.

**Table 2:** Smoking characteristics and reasons for smoking among smokers (n = 83). Values are presented as frequency (percentage) among smokers (n = 83). Percentages were calculated based on the total number of smokers. Smoking partner indicates whether the participant typically smokes with another person. Tobacco use in household refers to whether tobacco use occurs within the participant’s household environment. Associations between categorical variables and reasons for smoking were evaluated using the Chi-square test (χ²). The corresponding test statistic values (χ²) and p-values are reported. A p-value < 0.05 was considered statistically significant.

Variable	Category	n (%)	Stress	Pleasure	Peer pressure	Statistical test	Test statistic (χ²)	p-value	Significance
Age of initiation	Under 18 years	45 (54.2)	18	18	9	Chi-square	χ² = 2.628	0.269	Not significant
	18 years and above	38 (45.8)	18	9	11				
Year of study	Preclinical phase	65 (78.3)	27	23	15	Chi-square	χ² = 1.113	0.573	Not significant
	Clinical phase	18 (21.7)	9	4	5				
Smoking partner	Yes	64 (77.1)	24	21	19	Chi-square	χ² = 5.858	0.053	Borderline significance
	No	19 (22.9)	12	6	1				
Tobacco use in household	Not allowed	54 (65.1)	24	14	16	Chi-square	χ² = 4.077	0.13	Not significant
	Allowed	29 (34.9)	12	13	4				

Association between FTND scores and participant characteristics

As shown in Table 3, nicotine dependence among smokers was assessed using the FTND and summarized using median scores and IQR.

**Table 3:** Association between FTND scores and selected variables among smokers (n = 83). Nicotine dependence scores are presented as median (IQR) among smokers (n = 83). The FTND score ranges from 0 to 10, with higher scores indicating greater nicotine dependence. Comparisons between two groups were performed using the Mann-Whitney U test, while comparisons across three or more groups were conducted using the Kruskal-Wallis test. Corresponding test statistics (U and H) and p-values are reported. A p-value < 0.05 was considered statistically significant. FTND: Fagerström Test for Nicotine Dependence.

Variable	Category	n	Median FTND score (IQR)	Statistical test	Test statistic	p-value	Significance
Gender	Male	42	4.0 (3.0-6.0)	Mann-Whitney U	U = 476.0	<0.001	Significant
	Female	41	2.0 (0.0-4.0)				
Guilt about smoking	Yes	58	3.0 (2.0-5.5)	Mann-Whitney U	U = 623.0	0.309	Not significant
	No	25	3.0 (0.0-5.0)				
Attempted to stop smoking (past 12 months)	Yes	62	4.0 (1.0-6.0)	Mann-Whitney U	U = 555.0	0.312	Not significant
	No	21	3.0 (0.0-5.0)				
Stress affecting smoking frequency	Yes	72	3.5 (1.0-6.0)	Mann-Whitney U	U = 287.0	0.141	Not significant
	No	11	2.0 (0.0-4.0)				
Year of study	Foundation year	7	3.0 (1.5-5.0)	Kruskal-Wallis	H(5) = 4.384	0.496	Not significant
	Year 1	15	2.0 (0.5-5.0)				
	Year 2	26	4.0 (2.0-6.0)				
	Year 3	17	3.0 (0.0-5.0)				
	Year 4	12	3.0 (1.0-5.5)				
	Year 5	6	1.0 (0.0-2.5)				
Age at smoking initiation	Under 14 years	11	4.0 (3.0-7.0)	Kruskal-Wallis	H(2) = 3.898	0.142	Not significant
	15-17 years	34	2.5 (1.0-5.0)				
	18 years and above	38	3.0 (0.0-5.0)				
Number of tobacco products used	1	27	3.0 (1.0-5.0)	Kruskal-Wallis	H(5) = 12.336	0.03	Significant
	2	29	2.0 (0.0-4.0)				
	3	15	4.0 (2.5-5.0)				
	4	3	6.0 (4.0-6.0)				
	5	7	7.0 (2.0-8.0)				
	6	2	8.0 (8.0-8.0)				

A statistically significant difference in FTND scores was observed between genders (Mann-Whitney U test, U = 476.0, p < 0.001), with male smokers demonstrating higher median nicotine dependence scores compared to female smokers. No statistically significant differences in FTND scores were observed in relation to feelings of guilt about smoking (U = 623.0, p = 0.309), attempts to stop smoking during the past 12 months (U = 555.0, p = 0.312), or perceived stress affecting smoking frequency (U = 287.0, p = 0.141). Similarly, FTND scores did not differ significantly across year of study (Kruskal-Wallis test, H(5) = 4.384, p = 0.496) or age at smoking initiation (H(2) = 3.898, p = 0.142).

However, a statistically significant difference in FTND scores was observed based on the number of tobacco products used (Kruskal-Wallis test, H(5) = 12.336, p = 0.030), with higher dependence scores observed among participants using multiple tobacco products.

Participants' awareness regarding the health effects of smoking

Smokers’ awareness of the health consequences of smoking is summarized in Table 4. The most commonly identified health risk associated with smoking was lung cancer (19.9%), followed by lung disease (18.5%), heart disease (17.6%), and stroke (17.3%). Fewer participants identified chronic obstructive pulmonary disease (10.3%), while smaller proportions recognized other conditions such as pneumonia or oral cancer (4.0% each). Additionally, a minority of participants incorrectly identified rickets (vitamin D deficiency) as a consequence of smoking (4.5%).

**Table 4:** Participants’ awareness of the health effects of smoking among smokers (n = 83) Participants could select more than one response; therefore, percentages do not sum to 100%. Values are presented as frequency (percentage), and percentages were calculated based on the number of smokers who responded to this question (n = 83).

Health condition identified as a consequence of smoking	n (%)
Lung cancer	75 (19.9)
Lung disease	70 (18.5)
Heart disease	66 (17.6)
Stroke	65 (17.3)
Chronic obstructive pulmonary disease (COPD)	39 (10.3)
Rickets (vitamin D deficiency)	17 (4.5)
Pneumonia	15 (4.0)
Oral cancer	15 (4.0)
Other responses	10 (2.7)

Among smokers, 58 (69.9%) reported feeling guilty about smoking, 62 (74.7%) reported attempting to stop smoking during the past 12 months, and 72 (86.7%) reported that stress affected their smoking frequency.

## Discussion

This study set out to explore smoking behavior among health science students in the UAE, and the results show that smoking is still relatively common in this population, even though these students generally understand the health risks. The overall prevalence of 20.4% is similar to what other studies in the UAE have reported [4,7], which suggests that the problem is still ongoing and possibly not improving despite increased awareness efforts. Globally, tobacco remains a major preventable cause of disease [1,3], so seeing these numbers among future healthcare providers is concerning.

One of the more noticeable findings in our study was the significant difference between male and female students. Male students smoked significantly more, which is consistent with other research in the UAE [8]. This difference may reflect social norms that make smoking more socially acceptable for males. Surprisingly, medical students, who arguably receive the most in-depth training about the harms of tobacco, had higher smoking rates than students in other health majors. Similar trends have been observed elsewhere, where being knowledgeable does not necessarily stop students from smoking [11,12]. This could mean that stress or academic pressure is playing a bigger role than information alone.

A considerable proportion of smokers reported starting before the age of 18. Even though regulations exist, it seems that adolescents are still able to access tobacco products easily [3]. Another important finding is the growing popularity of vaping among students, as many students chose vaping more than traditional cigarettes or shisha, which is something that regional studies have also noted [4,7]. This may be due to the perception that vaping is less harmful, more discreet, or more socially acceptable, although the evidence does not necessarily support these assumptions.

Stress was the most commonly reported reason for smoking among participants, and many indicated that they smoke more when they feel stressed. This finding aligns with studies showing that stress is strongly linked to smoking habits among university students [9]. Peer influence was another strong factor, as students who had smoking partners were far more likely to smoke, which is consistent with behavioral theories explaining that social environments greatly influence health behaviors during young adulthood.

These findings can be interpreted within established behavioral frameworks such as the Theory of Planned Behavior and Social Cognitive Theory [14]. These models suggest that health behaviors are influenced by perceived social norms, environmental factors, and individual coping mechanisms such as stress management. This may help explain the observed associations between stress, peer influence, and smoking behavior among health science students.

As for nicotine dependence, it was higher among males and increased with multi-product use. This pattern shows that students who use more than one tobacco product tend to develop stronger dependence, which matches findings about shisha and cigarette dependence in other studies [9]. This is particularly concerning because multi-product use is becoming more common with the rise of vaping and other easily accessible tobacco products.

Even though awareness levels were generally high among respondents assessed in this study, it was clear that not all information was accurate. For example, misconceptions such as the incorrect association of smoking with rickets highlight gaps in detailed health knowledge despite generally high awareness levels. Similar findings have been previously reported [4], where awareness existed but was not always comprehensive. The heavy reliance on social media for health information may be contributing to these inconsistencies. While many students said they had tried to quit during the past year, very few used formal cessation resources. Most preferred to rely on personal methods such as avoiding triggers or using alternatives. This reflects previous findings that students often avoid structured cessation programs [10]. This may be due to lack of awareness, accessibility issues, or reluctance to seek help, as reported in studies on cessation programs [15].

Overall, the results highlight a stark contradiction: health science students know the harms of tobacco and encounter many cases during their training that highlight the harmful effects of smoking, yet many still smoke. This raises concerns about their future role in counseling patients about tobacco cessation, as smoking behavior could affect their confidence or credibility [11]. Universities should consider developing tailored interventions, including awareness campaigns targeting misconceptions about vaping and structured stress-management programs, as these campaigns and programs may significantly increase smoking cessation [16].

Limitations

This study has several limitations. First, the use of convenience snowball sampling may have introduced selection bias and limits the generalizability of the findings, as the sample may not be fully representative of all health science students across the UAE, particularly those from non-participating institutions. This sampling approach may have led to the overrepresentation of more socially connected or health-aware students, potentially influencing the observed prevalence estimates. In addition, non-response bias may be present, as students who chose to participate may have differed systematically from those who did not. Second, the cross-sectional design precludes any inference of causality between smoking behavior and associated factors. Third, the reliance on self-reported data may have introduced recall bias and social desirability bias, particularly given the sensitive nature of smoking behaviors. Underreporting of smoking behaviors may have occurred, especially among female participants due to cultural and social factors. Finally, multivariable analysis was not performed, limiting the ability to identify independent predictors of smoking and to control for potential confounding variables. Despite these limitations, this study provides important multi-university data on smoking behaviors among health science students in the UAE.

## Conclusions

This study found that smoking remains prevalent among health science students in the UAE, despite generally high awareness of its health risks. Stress was the most commonly reported reason for smoking initiation, and a substantial proportion of smokers reported using multiple tobacco products, with electronic cigarettes being the most commonly used product. Although awareness of smoking-related harms was generally high, gaps in knowledge persisted in specific areas, particularly regarding newer tobacco products and misconceptions about smoking-related health effects. Stress, peer influence, early initiation, and the use of multiple tobacco products were associated with smoking behavior in this sample. These findings highlight the need for targeted, evidence-based smoking prevention and cessation strategies within health science education settings, including stress management support, peer-focused interventions, and improved education on the risks of all tobacco products. Nationwide studies using more representative sampling methods are needed to improve the generalizability of these findings and to further inform public health policy and university-based interventions.

## Appendices

Appendix 1

Study Questionnaire (Author-Developed Items)

Section 1: Demographic information

1. Are you 18 years or older?

Yes

No

2. What is your gender?

Male

Female

3. What is your major?

Medicine

Dentistry

Pharmacy

Nursing

Physiotherapy

Medical Laboratory Sciences

Medical Diagnostic Imaging

Health Service Administration

Environmental Health Sciences

Clinical Nutrition and Dietetics

4. What is your year of study?

Foundation Year (Medical and Dentistry students)

Year 1

Year 2

Year 3

Year 4

Year 5

5. Which university do you attend?

University of Sharjah

Ajman University

Mohammed Bin Rashid University (MBRU)

Khalifa University (KU)

Gulf Medical University

6. What is your current living arrangement?

Living alone

Living with family

Section 2: Smoking status and behavior

7. How do you describe yourself?

Current smoker

Ex-smoker

Non-smoker

Social smoker

(Social smokers are defined as individuals who report occasional or non-daily smoking.)

8. At what age did you start smoking?

Under 10 years

11-14 years

15-17 years

18 years or older

9. What type of tobacco products do you use? (Select all that apply)

Cigarettes

Vape

Water pipe (Hookah)

Medwakh

Cigars

E-cigarettes (iQOS)

10. What is your main reason for consuming tobacco products?

Peer pressure

Stress

Pleasure

Parental influence

Curiosity/experimentation

11. Do you think your level of stress affects your smoking frequency?

Yes

No

12. Do you have a smoking partner?

Yes, with friends

Yes, with family

No

13. What is the status of tobacco use in your household?

Not allowed

Allowed

Section 3: Awareness of health effects

20. Which of the following do you believe are health risks of smoking? (Select all that apply)

Lung cancer

Melanoma (skin cancer)

Heart disease

Stroke

Rickets (vitamin D deficiency)

Lung diseases

Type 2 diabetes

Crohn’s disease

Chronic obstructive pulmonary disease (COPD)

Liver cancer

21. Where did you obtain information about the dangers of smoking? (Select all that apply)

Social media (internet)

Personal experiences

Awareness campaigns

Family and friends

Health warnings on tobacco products

Section 4: Perceptions and behavioral responses

22. Do you feel guilty about smoking?

Yes

No

23. During the past 12 months, have you tried to stop smoking?

Yes

No

24. If yes, which of the following strategies did you use? (Select all that apply)

Physician support

Nicotine replacement therapy (e.g., patches)

Support groups

Avoiding triggers

Developing alternative habits
